# CONservative TReatment of Appendicitis in Children: a randomised controlled feasibility Trial (CONTRACT)

**DOI:** 10.1136/archdischild-2020-320746

**Published:** 2021-01-13

**Authors:** Nigel J Hall, Simon Eaton, Frances C Sherratt, Isabel Reading, Erin Walker, Maria Chorozoglou, Lucy Beasant, Wendy Wood, Michael Stanton, Harriet Corbett, Dean Rex, Natalie Hutchings, Elizabeth Dixon, Simon Grist, Esther M Crawley, Bridget Young, Jane M Blazeby

**Affiliations:** 1 University Surgery Unit, Faculty of Medicine, University of Southampton, Southampton, UK; 2 Department of Paediatric Surgery and Urology, Southampton Children's Hospital, Southampton, UK; 3 UCL Great Ormond Street Institute of Child Health, London, UK; 4 Department of Public Health, Policy and Systems, Institute of Population Health, University of Liverpool, Liverpool, UK; 5 Primary Care, Population Sciences and Medical Education, Faculty of Medicine, University of Southampton, Southampton, UK; 6 Great Ormond Street Hospital for Children NHS Foundation Trust, London, UK; 7 Southampton Health Technology Assessment Centre, Faculty of Medicine, University of Southampton, Southampton, UK; 8 Centre for Academic Child Health, Bristol Medical School: Population Health Sciences, University of Bristol, Bristol, UK; 9 National Institute of Health Research (NIHR), Research Design Service South Central, University of Southampton, Southampton, UK; 10 Department of Paediatric Surgery, Alder Hey Children’s NHS foundation Trust, Liverpool, UK; 11 Department of Paediatric Surgery, St George's University Hospitals NHS Foundation Trust, London, UK; 12 Southampton Clinical Trials Unit, University of Southampton, Southampton, UK; 13 Patient and Public Involvement Representative, Southampton, UK; 14 Centre for Surgical Research and NIHR Bristol Biomedical Research Centre, Population Health Sciences, Bristol Medical School, University of Bristol, Bristol, UK

**Keywords:** therapeutics, qualitative research, gastroenterology, health services research

## Abstract

**Objective:**

To establish the feasibility of a multicentre randomised controlled trial to assess the effectiveness and cost-effectiveness of a non-operative treatment pathway compared with appendicectomy in children with uncomplicated acute appendicitis.

**Design:**

Feasibility randomised controlled trial with embedded qualitative study to inform recruiter training to optimise recruitment and the design of a future definitive trial.

**Setting:**

Three specialist paediatric surgery centres in the UK.

**Patients:**

Children (aged 4–15 years) with a clinical diagnosis of uncomplicated acute appendicitis.

**Interventions:**

Appendicectomy or a non-operative treatment pathway (comprising broad-spectrum antibiotics and active observation).

**Main outcome measures:**

Primary outcome measure was the proportion of eligible patients recruited. Secondary outcomes evaluated adherence to interventions, data collection during follow-up, safety of treatment pathways and clinical course.

**Results:**

Fifty per cent of eligible participants (95% CI 40 to 59) approached about the trial agreed to participate and were randomised. Repeated bespoke recruiter training was associated with an increase in recruitment rate over the course of the trial from 38% to 72%. There was high acceptance of randomisation, good patient and surgeon adherence to trial procedures and satisfactory completion of follow-up. Although more participants had perforated appendicitis than had been anticipated, treatment pathways were found to be safe and adverse event profiles acceptable.

**Conclusion:**

Recruitment to a randomised controlled trial examining the effectiveness and cost-effectiveness of a non-operative treatment pathway compared with appendicectomy for the treatment of uncomplicated acute appendicitis in children is feasible.

**Trial registration number:**

ISRCTN15830435.

**What is already known on this topic?**

- Non-operative treatment is an alternative to appendicectomy for children with uncomplicated acute appendicitis but the comparative effectiveness of these treatments is not known.
- A randomised trial comparing these treatments will be challenging due to the urgent care setting, complex treatments interventions and patient and surgeon preferences.
- Feasibility studies can help establish the feasibility of future RCTs and enhance their design to optimise chances of success and minimise waste of resources.

**What this study adds?**

- A future RCT comparing the effectiveness and cost-effectiveness of non-operative treatment with appendicectomy for children with uncomplicated acute appendicitis is feasible.
- Supported by embedded qualitative research to inform communication and recruiter training, we have identified opportunities to improve design and implementation of the future RCT.
- Hard to do trials where potentially eligible children often attend at night are feasible and acceptable particularly with iterative training for recruiters.

## Introduction

Acute appendicitis is the most common surgical emergency in children and is most frequently treated with appendicectomy. Recently non-operative treatment of appendicitis (with antibiotics alone and without an appendicectomy) has come to the fore as an alternate treatment approach but has not yet entered mainstream clinical practice. Barriers to the more widespread adoption of non-operative treatment include concerns over safety and efficacy and the fact that many surgeons are unfamiliar with this treatment strategy. Although appendicectomy is a tried and tested treatment, is familiar to surgeons and has a well-understood safety and efficacy profile, it does require a general anaesthetic and an abdominal operation with inherent risks. Many parents find the proposal that their child needs emergency surgery frightening and one they are keen to avoid if a viable alternative is available. While the existing evidence supports the safety of non-operative treatment,^1^ there is an evidence gap preventing meaningful comparison of the effectiveness and cost effectiveness of these treatment approaches;^2^ in particular, there is a lack of well-designed randomised controlled trials (RCTs) comparing appendicectomy with non-operative treatment.

In order to conduct an adequately powered pragmatic RCT, we first identified the need for further information on the feasibility of recruiting children to a RCT comparing appendicectomy with non-operative treatment. Issues to consider include: a lack of experience with non-operative treatment of acute appendicitis in the UK, so that surgeons may not be willing to recruit, and a challenging trial recruitment profile. Specifically, such a trial would involve recruiting children presenting acutely (often out of normal working hours), explaining a trial that has two very different treatment pathways in a time-limited setting, and relying on clinical staff with little trials experience to recruit to the trial in an unscheduled care setting. Furthermore, we identified several uncertainties regarding the design of a definitive effectiveness trial, not least the selection of appropriate primary and secondary outcomes.

We therefore performed a feasibility RCT with an embedded qualitative study. The aims were (i) to test feasibility of recruitment to an RCT of acute uncomplicated appendicitis in children in the UK, from both a patient and surgeon perspective; (ii) to optimise recruitment into the trial; (iii) to confirm the performance and safety of treatment pathways and (iv) to inform the design of a future definitive RCT. The detailed methods and results of the embedded qualitative study are reported elsewhere.^3^ The feasibility RCT was part of the CONservative TReatment of Appendicitis in Children randomised controlled Trial (CONTRACT) (feasibility) study that also included the development of a core outcome set for research involving children with uncomplicated appendicitis to identify outcomes important to a range of stakeholders, and a health economic feasibility study (both reported elsewhere).^4–6^ Here, we report key outcomes from the feasibility trial, which inform the viability of a future RCT, and key related findings from the embedded qualitative study.

## Methods

This feasibility RCT comparing appendicectomy and non-operative treatment in children with uncomplicated acute appendicitis was conducted in three specialist paediatric surgery centres in England and in accordance with a publicly available protocol.^7^

### Participants

Children aged >3 and<16 years with a clinical diagnosis, with or without radiological assessment, of uncomplicated acute appendicitis which prior to study commencement would be treated with appendicectomy, were eligible for inclusion. Exclusion criteria were: clinical signs or radiological findings to suggest perforated appendicitis; presentation with appendix mass; previous episode of appendicitis or appendix mass treated non-operatively; major anaesthetic risk precluding allocation to the appendicectomy arm; a known antibiotic allergy preventing allocation to non-operative treatment arm; antibiotic treatment started at referring institution (defined as two or more doses administered); cystic fibrosis; a positive pregnancy test or current treatment for malignancy. Of note, there was no requirement for diagnostic imaging in this study since imaging is not routinely used in the diagnostic evaluation of children suspected to have appendicitis in the UK^8 9^ and we wished to replicate current diagnostic pathways and deliver a pragmatic trial as far as possible.

### Participant identification, recruitment, randomisation

Participants were identified by the clinical team at the time of diagnosis and eligibility was confirmed by the research team as soon as possible. Eligible patients were approached by the treating clinical teams with support from dedicated research nurses. Potential participants were provided with written information about the study and shown a short video describing the study (available at http:/tinyurl.com/contract-f). From the time of first discussing the trial with potential participants and their families a maximum of 4 hours was permitted before a decision could be made regarding participation. This was to ensure there was no delay in providing treatment as a result of considering trial participation.

After written informed consent (and assent from children aged 12 years or over who wished to give it), a member of the trial team randomised the participant to one of two treatment groups in a 1:1 ratio via an independent web-based system, minimising for recruiting centre, sex (male/female), age (4–8/9–15) and duration of symptoms (onset of pain to recruitment into study) (<48 hours/≥48 hours), with complete prerandomisation concealment of treatment allocation. Participants were informed of their treatment allocation immediately.

### Interventions

#### Non-operative treatment arm

Children randomised to non-operative treatment were treated according to a clinical pathway designed specifically for this trial: fluid resuscitation, a minimum of 24 hours broad spectrum intravenous antibiotics (per local antimicrobial policy), a minimum of 12 hours nil by mouth (NBM) and regular clinical review to detect signs and symptoms of significant clinical deterioration (including, but not limited to, increasing fever, increasing tachycardia and increasing tenderness). After the initial 12-hour period of NBM, oral intake was advanced as tolerated. Children successfully treated without an operation were converted to oral antibiotics (per local policy) once they are afebrile for 24 hours and tolerating oral intake.

Clinical reviews were completed at approximately 24 and 48 hours postrandomisation. Any child who showed signs of significant clinical deterioration by 24 hours, or at any point during the trial, wase treated with appendicectomy. Children who were considered stable or improving continued with non-operative treatment. At 48 hours, any child who had not shown clinical improvement was scheduled for an appendicectomy. The decision to continue non-operative treatment at these time points or to recommend discontinuation of non-operative treatment and appendicectomy was made by the treating consultant based on clinical judgement. All reasons for change in treatment were recorded in detail in order to guide a clinical pathway in a future trial.

Any child who received an appendicectomy for an incomplete response to non-operative treatment, followed a standardised postoperative treatment regime already in use at each institution and identical to that used in the appendicectomy arm. The reason for having an appendicectomy was recorded.

Children treated non-operatively received a total of 10 days antibiotics following randomisation unless decided otherwise by the treating clinician. Children who received non-operative treatment were not offered interval appendicectomy but were counselled about the risk of recurrence.

#### Appendicectomy treatment arm

Children randomised to the appendicectomy arm underwent either open or laparoscopic appendicectomy at the surgeon’s discretion, performed by a suitably experienced trainee (as per routine current practice) or a consultant. Participants received intravenous antibiotics from the time of diagnosis and were treated postoperatively with intravenous antibiotics according to existing institutional protocols. The duration of antibiotic therapy was not standardised due to anticipated variation in intraoperative findings and in response to treatment. The type of antibiotics used was identical to those used in the non-operative treatment arm within each centre. Any child failing to respond to first-line antibiotics was treated as clinically appropriate with a longer course of antibiotics or a change in antibiotic therapy determined by intraoperative swab or fluid culture. Postoperatively, children with uncomplicated acute appendicitis or a normal appendix did not routinely have a nasogastric tube or a urinary catheter. They received oral intake as tolerated after surgery.

**Figure 1:**
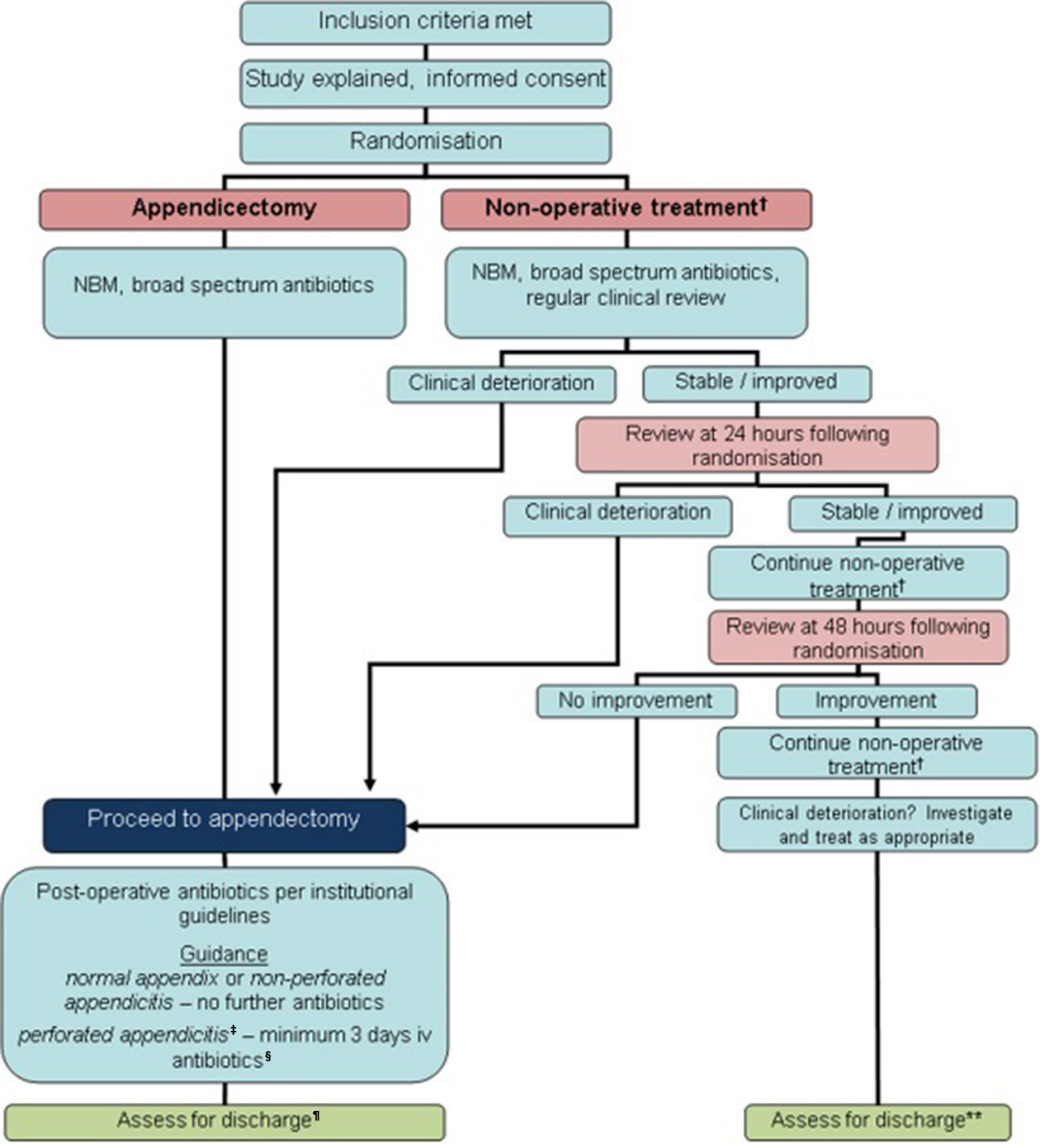
Clinical treatment pathway for each arm. *Appendicectomy group—no routine use of nasogastric tube or urinary catheter, advance diet as tolerates. †Non-operative treatment=NBM/sips for initial 12 hours minimum, then advance diet as tolerates; intravenous antibiotics 24 hours minimum, change to oral once afebrile for 24 hours, total course 10 days; analgesia. ‡Defined as either seeing a hole in the appendix or faecal matter/faecolith in the peritoneal cavity. §Continue intravenous antibiotics until afebrile for 24 hours, then change to oral; minimum 5 days total antibiotics. ¶Criteria for discharge include: vital signs within normal limits, tolerating light diet, adequate oral analgesia, mobile. NBM, nil by mouth.

The clinical pathway for the two treatment arms is shown in figure 1. The schedule of enrolment, interventions and follow-up has been previously reported.^7^

### Discharge assessment

Criteria for discharge home were the same in each treatment arm: vital signs within normal limits for age, afebrile for ≥24 hour, tolerating light diet orally, adequate oral pain relief and able to mobilise. Parents of all participants were given an information sheet at discharge informing them of ‘red flag’ symptoms and advised to contact their local unit if they had concerns about their child’s clinical progress.

### Follow-up and data collection

Participants were asked to complete a diary card for the first 14 days following hospital discharge about: medication taken (antibiotics and analgesia), whether they had been able to return to normal activity or full activity that day and if their parent(s) had had to take time off work because of their appendicitis. A clinic follow-up visit took place at 6 weeks with further trial follow-up at 3 and 6 months following discharge, either in the outpatient clinic or in the clinical research facility at each centre. If a face-to-face appointment was not possible, the 3-month and 6-month follow-up were completed by telephone. Data were recorded by dedicated research nurses at each site during hospital admissions and trial follow-up directly into an electronic, secure, web-based case report form.

### Primary outcome

The primary outcome was the recruitment rate into the trial defined as the proportion of eligible participants who were approached and recruited to the study over 12 months.

### Secondary outcomes

The secondary outcomes were:

1. Performance of study procedures including retention of participants for the duration of the study, and feasibility of outcome recording and data collection systems.
2. Willingness of parents, children and surgeons to take part in a randomised study comparing operative versus non-operative treatment and identify anticipated recruitment rate. This was assessed from audio recorded family-surgeon recruitment consultations, interviews with patients, parents, surgeons and nurses, surgeon surveys and focus groups.
3. Clinical outcomes of trial treatment pathways including (i) safety and overall success of initial non-operative treatment (measured as the number of patients randomised to non-operative treatment, discharged from hospital without appendicectomy); (ii) complications of disease and treatment (measured during hospital stay and 6-month follow-up period); (iii) rate of recurrent appendicitis during 6-month follow-up period. Further details of these outcomes including timing and method of measurement can be found in the published trial protocol.^7^

Blinding of treatment arm was not considered ethical or feasible, so treating staff, parents and participants were aware of treatment allocation. We aimed to determine the feasibility of a blinded discharge assessment and therefore blinded outcome assessment in a future RCT by attempting to complete a blinded discharge assessment for each participant. Once a decision to discharge the child had been made a member of the clinical team who had not been involved directly in the child’s treatment was asked to complete a discharge assessment. This assessor did not have prior knowledge of the randomisation or treatment received by the child. On completion of the discharge assessment, the assessor ‘guessed’ which treatment the child received. If the assessor became unblinded during the assessment, this was recorded.

### Sample size and statistical analysis

As a feasibility study, we did not specify a specific sample size but aimed to define our recruitment rate within an approximate 10% margin of error. Based on an anticipated study population available for recruitment of approximately 130 eligible participants across the three participating centres, we would be able to estimate a true 40% recruitment rate with a 95% CI of 31%–49% and a true 50% recruitment rate with a 95% CI of 41%–59%. These numbers of participants in the feasibility RCT would be adequate to test treatment pathway procedures, data collection methods and loss to follow-up. Accordingly, the study duration was defined *a priori* as being 12 months.

Data analysis was performed by the study statistician who was blinded to treatment allocation by the use of coded data. As this is a feasibility study, all analyses were treated as preliminary and exploratory, and data are reported descriptively. Feasibility outcomes (number of eligible patients, recruitment/retention rates, reasons for non-participation, success of blinding of the discharge assessor), treatment outcomes and complications are presented as simple summary statistics with 95% CI.

### Embedded qualitative study

Full methods and outcomes of this have been published elsewhere.^3^ The main aim of the qualitative study was to optimise trial recruitment on an iterative basis while the feasibility trial was still open to recruitment. Before recruitment started, we delivered generic communication training, informed by trial communication literature, to recruiting health professionals at all three trial sites. We subsequently developed bespoke communication training informed by the ongoing thematic analysis of the qualitative data, which comprised recruitment consultations and interviews with families and staff. Training was delivered to sites at the end of month 4 of recruitment and again during month 9 of recruitment. The potential impact of the qualitative study was evaluated by determining recruitment rates across three phases of the study: ‘phase one’ being the first 4 months (March to June 2017), ‘phase two’ months 5–9 and ‘phase three’ as the final 3 months.

### Trial governance

A Study Management Group was responsible for overseeing the day-to-day management of the trial. An independent Trial Steering Committee and Data Monitoring and Safety Committee were convened to provide oversight of the study. Their roles and responsibilities which included adverse event monitoring were agreed at the beginning of the trial and documented in specific charters. Specific processes to report adverse events in a timely manner to the relevant committee were agreed.

The trial was carried out in accordance with a published protocol that was developed in accordance with the SPIRIT-C guidance, was registered prior to recruitment of the first participant (ISRCTN 15830435) and has been published previously.^7^ The study is reported in accordance with the CONSORT 2010 statement (feasibility trial extension).^10^

### Changes to the study from the original protocol

Following an analysis of follow-up rates during the study, we introduced an incentive to improve follow-up rates and improve ascertainment of follow-up data. This was introduced in March 2018 following a successful application to the ethics committee to make this amendment. All participants who attended all remaining follow-up visits from that point onwards were offered a £10 voucher. The impact of this was assessed.

### Patient and public involvement

The methodology for this feasibility trial was developed with a study specific advisory group comprising young people and parents, many of whom had experience of acute appendicitis. These were invited to join this group through existing regional young person’s advisory groups and through contacts of the clinical investigators. This group met prior to the start of the study and again regularly throughout its duration. The group oversaw all aspects of the trial that involved interaction with patients and families including all patient and family facing documentation (eg, patient information sheets, consent forms, discharge information leaflets), the recruitment video, method and timing of initial approach for recruitment conversation and interview topic guides for the embedded qualitative research.

## Results

**Figure 2:**
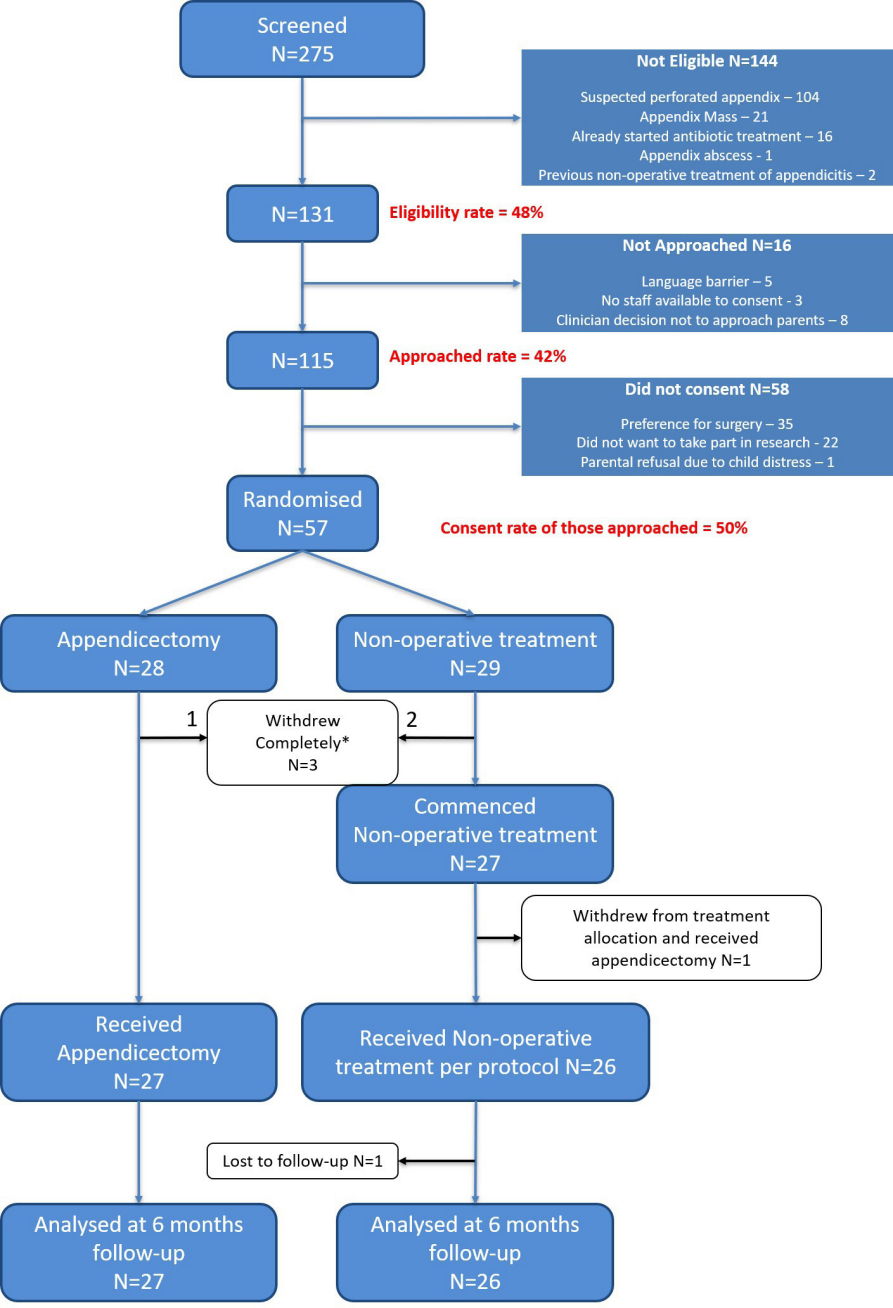
CONSORT diagram of CONTRACT feasibility RCT. *Indicates withdrew from allocated treatment and withdrew consent for further data collection and therefore not included in further reporting. CONTRACT, CONservative TReatment of Appendicitis in Children randomised controlled Trial.

All three centres opened to recruitment simultaneously on 1 March 2017 and were open to recruitment for 12 months until 28 February 2018. Final follow-up was completed on 31 August 2018. The trial profile is shown in figure 2.

### Recruitment and participants

Of 275 children screened, 131 fulfilled the eligibility criteria of whom 115 were approached about the study and of these 57 agreed to participate and were randomised. Thus, the primary outcome (proportion of eligible patients who were approached and recruited) was 44% (95% CI 35 to 52), and recruitment rate of those approached was 50% (95% CI 40 to 59). Despite anticipated challenges in recruitment, participants were successfully recruited into the trial outside of normal working hours (40% were recruited between the hours of 18:00 and 08:00 hours), recruitment rate per site across the whole study period ranged from 45% to 56% of those approached, and a total of 21 different surgeons successfully recruited participants. Of 57 participants randomised, baseline characteristics were well matched (table 1).

**Table 1:** Baseline characteristics of participants at randomisation

	Appendicectomy (n=28)	Non-operative treatment (n=29)	Total (n=57)
Age	10 years 7 months (6 years 4 months to 13 years 6 months)	10 years 3 months (5 years 0 months to 15 years 11 months)	10 years 5 months (5 years 0 months to 15 years 11 months)
Sex M:F (n)	18:10	18:10*	36:20*
Duration of symptoms (hours)†	32 (12–63)	34 (12–79)	33 (12–79)
US during diagnostic workup‡ (n (%))	8 (29%)	8 (28%)*	16 (28%)
Alvarado score†,§	5 (3–8)	5 (3–8)	5 (3–8)

Data are median (range) unless otherwise specified.

*Data for one participant who withdrew after randomisation not available.

†Note that there were some missing data for duration of symptoms and Alvarado score; for duration of symptoms, data are missing for 10 in appendicectomy arm and 8 (including one withdrawal) in non-operative treatment arm; for Alvarado score, data are missing for five (including one withdrawal) in non-operative treatment arm.

‡No child had a CT scan as part of diagnostic workup.

§The Alvarado score^22^ is a tool used to assess the likelihood of appendicitis in children presenting with abdominal pain. It was calculated for participants to provide a quantitative measure of severity of illness but was not used to determine eligibility for the trial or as a minimisation criteria.

US, ultrasound.

### Interventions received

Figure 2 shows participant flow through the clinical trial. Three participants withdrew consent for the study soon after randomisation including consent for continued data collection. They were therefore excluded from any further analysis. Reasons given for this were dissatisfaction with treatment allocated (n=2) and being too overwhelmed with the diagnosis to continue in the trial (n=1). The remaining 27 participants in the appendicectomy and 27 participants in the non-operative treatment arm both started the assigned intervention. All 27 participants in the appendicectomy received the allocated treatment whereas there was one protocol deviation in the non-operative treatment in a child whose parents withdrew consent for the study (but agreed to continued data collection) and requested appendicectomy 8 hours after randomisation.

In the appendicectomy arm, all 27 children received the allocated intervention and were treated according to the clinical pathway. Histological diagnosis of the resected appendix revealed uncomplicated acute appendicitis in 17 (63%), perforated appendicitis in 8 (30%) and a normal appendix in 2 (7%).

In the non-operative treatment arm, 19 of the 27 children (70%) responded successfully to non-operative treatment, followed the clinical pathway and were discharged from hospital. The remaining eight children underwent appendicectomy during their initial hospital admission. Reasons for appendicectomy were parental choice (withdrawal from treatment arm as described above) at 8 hours following randomisation (n=1), deterioration in clinical condition (according to protocol) at range 12–44 hours following randomisation (n=6) and no improvement after 48 hours of non-operative treatment (in accordance with protocol, n=1). In the children who deteriorated (n=6), clinical reasons given included persistent fever and pain, worsening peritonism and worsening pain. In the one child who did not have any symptomatic improvement after 48 hours, the primary persisting complaint was that of ongoing severe abdominal pain. All these eight patients underwent successful appendicectomy during the initial hospital admission. Histological findings were uncomplicated acute appendicitis in four and perforated appendicitis in four (all of whom had deteriorated during initial non-operative treatment).

### Discharge assessment and follow-up

Thirty-four of 54 eligible children (63%) underwent a blinded discharge assessment. In the remaining 20, this was not possible due to non-availability of appropriate members of staff. In one case, the assessor was unblinded during the assessment. Following discharge, 26 of 54 (48%) participants (15 in the appendicectomy arm and 11 in the non-operative treatment arm) returned a diary card, the majority of which (n=23, 88%) were completed in full. One child was completely lost to follow-up and did not attend any follow-up appointment or could be contacted by phone. This child was withdrawn from the study after the 3-month follow-up timepoint as they were known to have moved overseas. The remaining participants attended follow-up appointments or were contacted by phone at 6 weeks (n=48/54, 89% of those remaining in the study), 3 months (n=46/54, 85%) and 6 months (n=45/53, 85%). All other participants either did not attend or did not respond to repeated requests for contact by telephone.

Part way through the study, we introduced an incentive of a £10 shopping voucher to participants who completed all future follow-up assessments. Of the 3-month follow-up appointments, 47 were not incentivised and were completed by 39 participants (83%, 95% CI 72% to 93%), whereas 7 were incentivised and all 7 were completed (100%, 95% CI 59% to 100%). Of the 6-month follow-up appointments, 34 were not incentivised and were completed by 28 participants (83%, 95% CI 65% to 93%) and 19 were incentivised and were completed by 17 participants (89%, 95% CI 67% to 99%).

### Secondary outcomes

There was no mortality in the 6 months following enrolment. During the 6-month follow-up, seven children in the non-operative treatment arm developed recurrent appendicitis (24% of those randomised to non-operative treatment; 37% of the 19 who initially responded to non-operative treatment). Six of the seven underwent appendicectomy, with histological findings of simple acute appendicitis (n=4) and perforated appendicitis (n=2). The remaining participant presented with an appendix mass at the time of recurrence, which was successfully treated non-operatively and subsequently underwent interval laparoscopic appendicectomy. At the final follow-up, 11 children initially randomised to receive non-operative treatment (41%) had not undergone appendicectomy. In the appendicectomy arm, three children (11%) were readmitted to hospital following initial treatment for treatment with intravenous antibiotics for fever and abdominal pain (n=1) or intra-abdominal fluid collection/abscess (n=2) including one child also treated with percutaneous drainage.

Adverse events during the 6-month follow-up are summarised in table 2. In the appendicectomy arm, 22 adverse events were reported in eight participants and in the non-operative treatment arm, 24 adverse events were reported in 15 participants. Of these, a small number were assigned as ‘possibly’ or ‘definitely’ attributed to the study intervention including: a rash at the time of receiving antibiotics (n=2), wound complications (dehiscence x1, infection x1, suture complications x1) and intra-abdominal fluid collections treated with drainage and antibiotics (n=1) or antibiotics alone (n=1).

**Table 2:** Adverse event (AE) profile of each treatment group

Subject	AE description	AE actions	AE severity	AE serious*	AE related to treatment arm	AE outcome
**A: non-operative treatment group**						
009	Fever-readmission	Further course of antibiotics given	Moderate	Yes*	Definitely	Resolved with sequelae
	PICC line insertion	Weekly follow up appointments in clinic	Moderate	No	Unrelated	Resolved
025	Abdominal pain	–	Moderate	Yes*	Unrelated	Resolved
	Sore throat	Ibuprofen given	Mild	No	Unrelated	Resolved
103	Abdominal pain	Antibiotics	Moderate	Yes*	Unlikely	Resolved
	Recurrent appendicitis	Appendicectomy	Severe	Yes*	Unlikely	Resolved
162	Abdominal pain	Appendicectomy and hospital admission	Moderate	No	Definitely	Ongoing
	Fluid collection	Continued antibiotics. Already in hospital	Moderate	No	Definitely	Ongoing
179	Abdominal pain	A&E attendance, bloods taken	Mild	No	Unrelated	Resolved
233	Abdominal pain	Appendicectomy and hospital admission	Moderate	No	Definitely	Resolved
	Recurrence of appendicitis	Appendicectomy	Moderate	No	Definitely	Resolved
266	Recurrent appendicitis	Appendicectomy	Moderate	No	Definitely	Resolved
002	Abdominal pain	Admission to hospital	Mild	No	Probably	Resolved
157	Abdominal pain	–	Moderate	No	Possibly	Resolved
049	Abdominal pain	Advice given in A&E	Mild	No	Unrelated	Resolved
089	Patient visited GP surgery complaining of being sleepy for 2 weeks	None	Mild	No	Possibly	Ongoing
	Patient visited GP complaining of lethargy for 2 weeks	None	Mild	No	Possibly	Ongoing
	Abdomen pain	Patient had appendicectomy	Moderate	No	Probably	Resolved
184	Abdominal pain	Patient to be sent a clinic appointment	Mild	No	Unlikely	Ongoing
185	Rash over thighs	Patient given three doses of antihistamine	Mild	No	Unrelated	Resolved
276	Lethargic	Attended A+E. Discharged. Seen in clinic	Mild	No	Probably	Resolved
289	Generalised rash	Started antihistamines	Mild	No	Possibly	Resolved
**B: Appendicectomy**						
014	Abdominal pain	Intravenous antibiotics	Moderate	Yes	Possibly	Resolved
	Fever postop on readmission	NA	Mild	No	Possibly	Resolved
017	Vomiting	Ultrasound scan	Moderate	Yes	Unlikely	Resolved
123	Headache	Paracetamol given	Mild	No	Unrelated	Resolved
	Abdominal pain	Blood test	Moderate	No	Possibly	Resolved
	Intermittent vomiting	Blood test taken on 06/11/2017 was fine	Moderate	No	Possibly	Resolved
	Headache	Ibuprofen, time off school	Mild	No	Possibly	Resolved
	Sickness	Rest, off sick from school	–	No	Possibly	Resolved
	Sickness	Surgical review	Mild	No	Unrelated	Resolved
	Sickness	Off school	Mild	No	Unrelated	Resolved
264	Fluid collection	Drain insertion and hospitalisation	Moderate	No	Unrelated	Resolved
	PICC line insertion	Hospitalisation	Moderate	No	Unrelated	Resolved
	Drain insertion	Hospitalisation	Moderate	**No**	Unrelated	Resolved
040	Localised intra-abdominal fluid collection	Treatment with intravenous antibiotics	Moderate	No	Possibly	Resolved
167	Inflamed wound site	Patient started oral flucloxacillin	Moderate	No	Definitely	Resolved with sequelae
	Wound dehiscence	Attended accident and emergency	Moderate	No	Definitely	Resolved
	Diarrhoea	Telephone consultation with GP	Mild	No	Unlikely	Resolved with sequelae
	Vomiting	Call to GP—stomach bug diagnosed	Mild	No	Unrelated	Resolved
	Diarrhoea	Call to GP—stool sample	Mild	No	Unrelated	Resolved
	Pharyngitis	Started oral amoxicillin	Mild	No	Unrelated	Resolved
245	Wound infection	Started antibiotics	Moderate	No	Definitely	Resolved
247	Suture-related complication	Ultrasound and clinic appointment	Moderate	No	Probably	Resolved

PICC, peripherally inserted central venous catheter; GP, general practitioner.

*Note that AEs that were reported as serious (SAEs) during the early months of the trial are included here as reported on the basis of standardised reporting terminology in RCTs in that they resulted in either prolongation of hospital stay, readmission to hospital or death. However, since these were all predictable and related more to the disease process rather than the study interventions, we subsequently reclassified these ‘expected’ SAEs as AEs in protocol amendment 1 10 April 2017. Thus, although similar AEs did occur beyond the first 2 months of the study, they were no longer reported as SAEs.

### Embedded qualitative study

The findings of the qualitative study are reported elsewhere.^3^ These findings shaped the first bespoke training session at the end of month 4, which focused on encouraging health professionals to use terminology that conveyed equipoise, exploring family treatment preferences and providing balancing information and supported health professionals in responding to frequently asked questions from families. The second bespoke communication training session in month 9 (again informed by qualitative study findings) focused on ways that surgeons had improved their communication about CONTRACT since the first training, particularly with regard to equipoise. We also revisited examples of non-optimal communication as well as ways to enhance explanations of randomisation, manage families’ expectations about scheduling of surgery if non-operative treatment failed, provide balanced communication of treatment risks, (ie, discuss risks relating to both treatment arms) and respond to families’ further questions (see full report in Ref. 3). Recruitment rate increased following each of these bespoke training sessions from 38% during the initial 4 months to 47% in months 5–9 and to 72% in months 10–12. Final recommendations from the qualitative study to inform the design of a future definitive trial, and paediatric urgent care surgical trials more broadly, are summarised in box 1.

Box 1 **Recommendations to optimise informed consent, recruitment and retention in future paediatric urgent care surgical trials**

1. Involve children and young people in research discussions and decision-making where possible, as per current guidance (1), while being sensitive to parents’ anxieties about what their children hear regarding treatment complications.
2. Provide families with advance information about how a child’s treatment will be managed prerandomisation and in both treatment arms. Where relevant, this should include the timing of trial treatments and the timeframe in which families should expect to see an improvement in their child’s conditions.
3. Parents may link treatment delays to the additional procedures required for the trial and this could discourage them from participating, or remaining, in the trial. Where possible, delays in delivering treatments prerandomisation and postrandomisation should be minimised.
4. In cases where children and parents differ in their treatment preferences, randomisation may offer a means of resolving this conflict. Sensitively explain treatment arm allocation to trial participants. If children are upset with their allocation, further exploring their treatment preference may help to allay their concerns.
5. Time to decide: develop a strategy to allow families to indicate when they have made a decision regarding participation, so minimising delays from the perspective of families.
6. Consider staffing strategies to support health professionals in recruiting families outside of normal working hours.
7. Be aware of family sensitivities when explaining postsurgery findings in the context of a trial.

## Discussion

To our knowledge, this is the first study to determine the feasibility of recruiting children with uncomplicated acute appendicitis to a RCT comparing appendicectomy with a non-operative treatment pathway. Our results show that it is possible to identify and recruit children with uncomplicated acute appendicitis even outside of normal working hours (an important consideration since children with appendicitis frequently present at night and at the weekend). We achieved this by integrating the research protocol with clinical teams. The study conduct was good too. Paediatric surgeons adhered to the randomised allocation and patients and families reported that trial pathways and follow-up were good and acceptable. The qualitative work identified training requirements of clinical teams and this allowed trial specific training packages to be developed to optimise communication and recruitment. Overall, our findings support proceeding to a full definitive RCT to determine the comparative effectiveness of appendicectomy and non-operative treatment.

The main strength of this study is that we have established that a future RCT is possible by successfully recruiting to a feasibility RCT. A further strength is that we demonstrated this in three geographically and sociodemographically distinct UK centres with three different groups of surgeons. Additionally, we embedded qualitative methodology to develop bespoke training for recruiting teams and implemented this to optimise communication and recruitment within the study. The principal limitation is that we have conducted the work only in three centres and these may not be representative of future sites. However, additional work we have completed with other sites suggests there is adequate interest to proceed with a larger trial and we will ensure adequate support and training for clinical and research teams at new sites for our future RCT including through the NIHR Clinical Research Network.

In keeping with previous studies that have evaluated recruitment of children to RCTs in urgent care settings, we found that parental uptake was adequately high to make a future trial feasible.^11 12^ Our study of children with acute appendicitis further contributes to this field and our findings likely have implications for the design and delivery of other urgent care trials in children even where trial interventions are complex. Our work provides evidence for child health researchers contemplating ‘hard to do’ trials where potentially eligible children often attend out of normal hours that such trials are feasible and acceptable particularly with iterative training for recruiters. Through this feasibility trial, we are able for the first time to report on the safety of non-operative treatment for uncomplicated acute appendicitis in the UK. We acknowledge other reports of this treatment modality from overseas.^13 14^ This is an important aspect given the lack of familiarity with this treatment approach among most UK surgeons and further supports proceeding to a larger RCT. While we acknowledge an assessment of safety of non-operative treatment based on the relatively small numbers here should be made with caution, our findings that there is no evidence of harm are in keeping with a growing body of literature suggesting that non-operative treatment is indeed a safe treatment approach.^2 15^ This finding is also timely, because more recently there has been a dramatic uptake of non-operative treatment for appendicitis during the SARS-CoV-2 pandemic in both adults and children.^16 17^ While there are several similar ongoing trials comparing non-operative treatment with appendicectomy for children with uncomplicated acute appendicitis,^18 19^ none are recruiting in the UK. We continue to believe that a UK trial is important to understand the comparative effectiveness of these two very different treatments and the comparative cost-effectiveness within the NHS. Given the increase in use of non-operative treatment associated with the SARS-CoV-2 pandemic,^17^ understanding the outcomes of these different treatment approaches is arguably now more important than ever.

The clinical features and outcomes of participants in this feasibility RCT are not the focus of this report but some aspects are worthy of discussion in relation to feasibility of a future trial. Baseline characteristics of trial participants were as anticipated from the existing literature, suggesting that we have enrolled a representative population.^8 20^ Despite our efforts to only enrol children with uncomplicated appendicitis, 30% of children allocated to receive appendicectomy had complicated appendicitis. Our experience suggests that differentiating between uncomplicated and complicated disease based only on clinical judgement by a paediatric surgeon is inadequate both for a future RCT and future clinical practice. Related to this is that we suspect the relatively low success rate of non-operative treatment reported here when compared with other studies^2^ is likely due to recruitment of children with more advanced appendicitis than had been intended. Given the well-matched baseline characteristics and randomised study design, we assume that a similar proportion in the non-operative treatment arm also had complicated appendicitis. For a future trial to be acceptable to all stakeholders therefore, it will be necessary to exclude those children with complicated appendicitis more reliably. Some similar studies have used ultrasound to make an assessment of severity of appendicitis and thereby eligibility.^13 18 21^ While seemingly attractive, the reliability of ultrasound is not 100% for this purpose and more importantly ultrasound is not routinely used for diagnostic purposes in the UK. In this trial, just 28% of participants received an ultrasound and in a recent large UK observational study, just 40% of children presenting with abdominal pain had an ultrasound.^8^ Any trial protocol that demands ultrasound to assess eligibility would limit the trial generalisability, limit the number available for recruitment and would not be representative of current UK practice. Instead, we intend to use a bespoke validated scoring system based on clinical and laboratory parameters; work is underway to develop this.

In conclusion, these data show that it is feasible to recruit children with uncomplicated acute appendicitis to a RCT comparing appendicectomy with non-operative treatment. There is no evidence for a poor safety profile of non-operative management. The comparative effectiveness of appendicectomy and non-operative treatment of uncomplicated acute appendicitis remains an important research question. The next step is to complete a definitive RCT with inclusion criteria modified to prevent recruitment of children with perforated appendicitis and this feasibility trial has been crucial in refining the design and implementation of that study.

## Data Availability

Data may be made available subject to reasonable request and subsequent executed data sharing agreement.
