# Correction: *CONservative TReatment of Appendicitis in Children: a randomised controlled feasibility Trial (CONTRACT)*

**DOI:** 10.1136/archdischild-2020-320746corr1

**Published:** 2021-10-18

Hall NJ, Eaton S, Sherratt FC, *et al*. CONservative TReatment of Appendicitis in Children: a randomised controlled feasibility Trial (CONTRACT). *Arch Dis Child* 2021;106:764–73. doi:10.1136/archdischild-2020-320746

This article has been corrected since it first published. The provenance and peer review statement has been included.

